# Short-Term Detraining Does Not Impair Strength, Speed, and Power Performance in Elite Young Soccer Players

**DOI:** 10.3390/sports8110141

**Published:** 2020-10-25

**Authors:** Lucas A. Pereira, Tomás T. Freitas, Bruno Pivetti, Pedro E. Alcaraz, Ian Jeffreys, Irineu Loturco

**Affiliations:** 1 NAR-Nucleus of High Performance in Sport, São Paulo 04753060, Brazil; lucasa_pereira@outlook.com (L.A.P.); tfreitas@ucam.edu (T.T.F.); 2 Department of Human Movement Sciences, Federal University of São Paulo, São Paulo 11015020, Brazil; 3 UCAM Research Center for High Performance Sport, Catholic University of Murcia, 30107 Murcia, Spain; palcaraz@ucam.edu; 4 Vitoria Sport Club, Salvador 41750240, Brazil; brunopivetti@yahoo.com.br; 5 Faculty of Sport Sciences, Catholic University of Murcia, 30107 Murcia, Spain; 6 University of South Wales, Pontypridd CF37 1DL, UK; ian.jeffreys@allproperformance.co.uk

**Keywords:** football, neuromuscular abilities, training interruption, team-sports, physical performance

## Abstract

This study aimed to examine the effects of short-term detraining on the strength, speed, and jump capacities of under-20 soccer players. Twenty-four elite under-20 soccer players from the same professional club were assessed pre and post 26 days of detraining. The measurements were performed in the following order: countermovement jump (CMJ); 10 m linear sprint velocity; and one-repetition maximum test (1RM) in the horizontal leg-press exercise. To analyze the differences between pre- and post-tests, a paired T-test was applied. The significance level was set as *p* < 0.05. Soccer players exhibited a significant increase in CMJ performance (*p* = 0.02) and no significant differences in 10 m sprint velocity and 1RM leg-press were found after the short-term training cessation (*p* = 0.61; *p* = 0.55, respectively). We demonstrated that a short-term detraining period was capable of promoting a significant increase in the vertical jump height without inducing negative effects on the strength and speed capabilities of elite under-20 soccer players. Practitioners and sport scientists should be aware of these findings to program more effective training strategies at the beginning of the subsequent training cycle.

## 1. Introduction

One of the greatest challenges for coaches is planning and organizing successful training periodization programs for professional athletes. During the preparation process, the organization of training content using distinct training phases is critical to the appropriate development of physical abilities designed to impact competitive performance [1,2,3]. Specifically, during the early periodization stages, more attention is paid to the improvement in basic physical capacities such as maximum strength and endurance, a period commonly called the “general preparation phase” [1,2,3,4]. In contrast, close to competitions, coaches usually emphasize the development of speed- and power-related qualities, during the so-called “specific preparation phase” [4,5,6]. Although several studies have examined the effects of training organization on sports performance, less attention has been given to a phase that athletes inevitably face at least one time per season: the detraining period.

Detraining can be defined as a partial reduction or total interruption in training loads, leading to a series of physical and physiological adaptations [7]. Previous studies have reported significant reductions in strength-power performance in athletes with different training backgrounds after long-term detraining (i.e., periods longer than 4 weeks) [8]. On the other hand, during short-term detraining (i.e., <4 weeks), the level of evidence is limited [7,9], with some studies demonstrating no changes or even increases in certain strength, speed, and power qualities after periods of inactivity [10,11,12]. For example, Hortobágyi et al. [10] did not observe changes in vertical jump height in resistance-trained men after a 2 week detraining period. Conversely, Loturco et al. [11] detected meaningful increases in the rate of force development and 5 and 15 m sprint velocity in elite women pole-vaulters after a detraining period of 28 days. Therefore, to date, it is not possible to determine whether short detraining phases can be beneficial or harmful for top-level athletes. A deeper understanding of this issue is fundamental for coaches and sport scientists, as it may affect the selection and sequencing of training content and intensity throughout the preparation period. This becomes even more critical in team-sports, where players usually have limited time for training during the initial phases of the year (i.e., training pre-seasons; that always occurs after short periods of detraining) [2,6,13].

Specifically, in soccer, athletes typically have ~3 or 4 weeks of training before starting the competitive season (i.e., pre-season) [4,6,13,14]. Soccer pre-seasons are increasingly characterized by reduced periods and very-congested schedules, both of which compromise the adequate preparation of players and, hence, their performance [6,13,15]. Considering the above-mentioned factors, it is important to examine the effects of short-term detraining (i.e., ≤4 weeks) on some specific physical capacities. In this context, it is essential to determine if “general preparation phases” are required (for example, when neuromuscular performance decreases) or not (when neuromuscular performance stabilizes or increases) after these short periods. This information can help coaches design effective in-season strategies, where the opportunities for specific physical and technical development are often limited. Thus, the purpose of this study was to analyze the effects of 26 days of detraining on the strength, speed, and jump abilities of under-20 soccer players. Based on previous studies that did not find significant differences in the neuromuscular performance of top-level athletes after short-term detraining [10,11], we hypothesized that the elite young soccer players would not exhibit significant changes in strength, speed, and jump capacities.

## 2. Materials and Methods

### 2.1. Participants

A convenience sample of twenty-four elite under-20 soccer players (age: 18.7 ± 0.4 years; height: 178.3 ± 5.4 cm; body-mass (BM): 70.9 ± 6.1 kg) from the same professional club, with at least six years of experience in a professional academy, participated in this study. Soccer players were assessed pre and post a detraining period of approximately 4 weeks. The team from which the players were drawn participated in the most important under-20 competitions of the country, attesting the high level of competition of the study participants. The research was approved by the Federal University of São Paulo (4.355.629) Ethics Committee and all participants signed an informed consent form before participating in the study.

### 2.2. Study Design

This descriptive longitudinal study assessed the variations in vertical jump, strength, and sprint velocity over 26 days of training interruption in elite under-20 soccer players. The physical assessments were performed after they participated in the *São Paulo Cup of Junior Soccer Players*, the most important under-20 tournament in Brazil, and before starting the preparation period for the *Paulista State Championship*. During this period, players were required to avoid any type of general or specific systematic training, being instructed to perform only light or moderate physical activities (e.g., walking and jogging). In addition, they followed a nutritional plan developed to help them to maintain their BM and body composition during the short-term detraining. To ensure that soccer players followed these recommendations, at the post-assessments, they were asked about their activities throughout this period and none of them violated the study protocol. The tests were performed on the same day and in the following order for all participants at the two testing times: (1) countermovement jump (CMJ); (2) 10 m linear sprint velocity; (3) one-repetition maximum (1RM) in the horizontal leg-press exercise. All players were well familiarized with testing procedures due to their regular assessments in our facilities. Before the tests, athletes performed a standardized warm-up including general (i.e., running at a moderate pace, rating “3” in the 0–10 rating of perceived exertion scale [16] which was assessed every 30-s, for 10 min followed by dynamic stretching for 3 min) and specific exercises to facilitate maximal performance (i.e., five progressive submaximal jumps and sprints). Between each test, a 15 min rest interval was provided to explain the procedures, allow adequate recovery, and adjust the equipment.

### 2.3. Experimental Procedures

#### 2.3.1. Body Mass

BM was measured using a digital scale with an accuracy of 0.1 kg (Filizola Industry, São Paulo, Brazil). The measurements were performed before the warm-up activity, with athletes barefoot and wearing training clothes.

#### 2.3.2. Countermovement Jump

Vertical jump height was determined using the CMJ. The soccer players were instructed to execute a downward movement followed by a complete extension of the legs. All attempts were executed with the hands placed on the hips. The CMJ was performed on a contact platform (Smart Jump, Fusion Sport, Brisbane, Australia). The validity and reliability of this measurement system have been previously demonstrated by Reeve and Tyler [17]. A total of five attempts were allowed, interspersed by 15 s. The best attempt was retained for data analysis purposes.

#### 2.3.3. Linear Sprint Test

Two pairs of photocells (Smart Speed, Fusion Sport, Brisbane, Australia) were positioned at the starting line and the 10 m distance. Soccer players sprinted twice at maximum speed, starting from a standing position, 0.5 m behind the starting line. Sprint velocity was calculated as the distance traveled over a measured time interval. A 5-min rest interval was allowed between the two attempts and the fastest time was considered. The sprint tests were performed indoors, on an artificial turf surface composed of polyethylene and monofilament fibers (~100 μm thick).

#### 2.3.4. One-Repetition Maximum in the Horizontal Leg-Press Exercise

Maximum dynamic strength was assessed using the leg-press exercise test as described previously [14,18]. Before the test, the subjects executed a specific warm-up set, which consisted of 5 repetitions at ~40–50% of the estimated 1RM followed by three repetitions at 70% of the estimated 1RM. A 3 min rest interval was provided between all sets. After 3 min, athletes started the test and were allowed up to five attempts to achieve their 1RM (i.e., maximum mass [kg] that could be lifted once using the proper technique) [18]. The measurements were performed on a Plyo Press machine (Plyo Press; Athletic Republic, Park City, UT, USA), and the participants started the concentric movement from 90° knee flexion. Strong verbal encouragement was provided during the attempts. Since no changes were observed in the subject’s BM comparing both periods of assessment, we opted to present the absolute 1RM values to facilitate their interpretation.

### 2.4. Statistical Analyses

The statistical analysis was performed with the SPSS^®^ software package version 22.0 (SPSS, Inc., Chicago, IL, USA). The normality of data was confirmed via the Shapiro–Wilk test. To analyze the pre-post differences in the variables tested, the paired *t*-test was applied. The significance level was set as *p* < 0.05. Additionally, the magnitudes of the differences were interpreted through Cohen’s *d* [19] effect sizes (ES) using the following thresholds: <0.2, 0.2–0.6, 0.6–1.2, 1.2–2.0, 2.0–4.0, and >4.0 for trivial, small, moderate, large, very large, and near-perfect, respectively [20]. All tests used here demonstrated small errors of measurement, as evidenced by their high levels of accuracy and reproducibility (i.e., coefficient of variation < 5% and intraclass correlation coefficient > 0.90 for all assessments) [20].

## 3. Results

**Figure 1:**
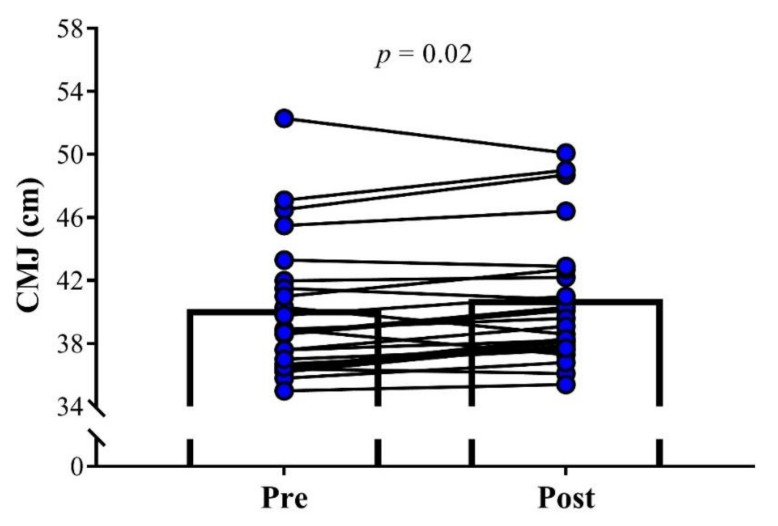
Comparison of the countermovement jump height (CMJ) pre and post 26 days of detraining in under-20 soccer players. The significance level was set as *p* < 0.05.

**Figure 2:**
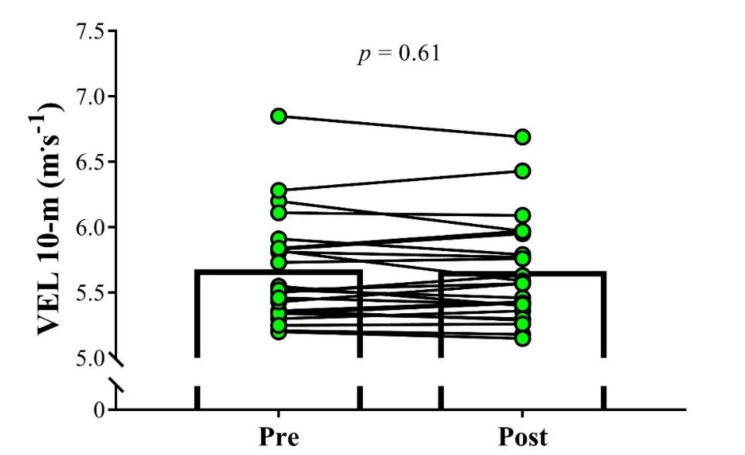
Comparison of the 10 m sprint velocity (VEL) pre and post 26 days of detraining in under-20 soccer players. The significance level was set as *p* < 0.05.

**Figure 3:**
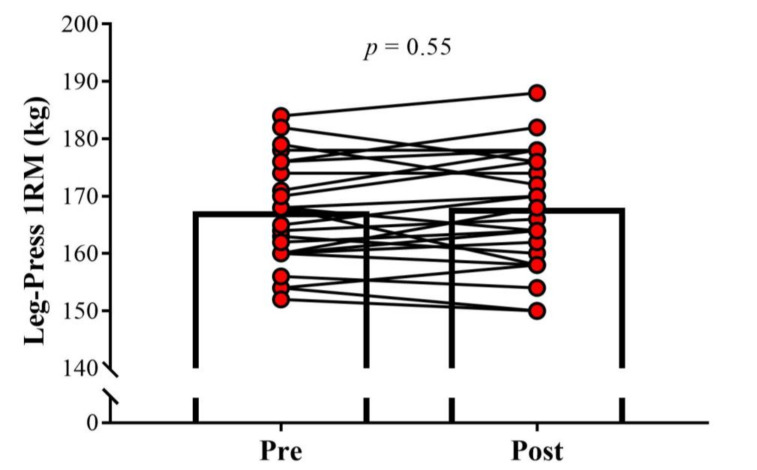
Comparison of the one-repetition maximum test (1RM) in the leg-press exercise pre and post 26 days of detraining in under-20 soccer players. The significance level was set as *p* < 0.05.

No significant change in the BM was observed between pre- and post-assessments (Pre: 70.9 ± 6.1 kg; Post: 71.1 ± 7.4 kg; *p* = 0.92, ES = 0.03). Soccer players demonstrated a significant increase in CMJ performance after the training cessation period (Pre: 40.0 ± 4.3 cm; Post: 40.6 ± 4.1 cm; *p* = 0.02; ES = 0.15; Figure 1). No significant differences were found after 26 days of detraining for 10 m sprint velocity (Pre: 5.66 ± 0.40 m·s^−1^; Post: 5.64 ± 0.39 m·s^−1^; *p* = 0.61, ES = 0.03, Figure 2) and 1RM leg-press (Pre: 166.8 ± 9.2 kg; Post: 167.4 ± 10.2 kg; *p* = 0.55, ES = 0.06, Figure 3).

## 4. Discussion

We investigated the variations in strength, speed, and jumping abilities of under-20 soccer players after approximately 4 weeks of training cessation. Our results revealed no significant changes in leg-press 1RM and 10 m sprint velocity; nonetheless, a significant increase was observed in CMJ height. These findings certainly impact training prescription after short periods of detraining. As a consequence, soccer coaches and sport scientists are advised to assess their players not only before and after specific training periods (e.g., pre-seasons), but also at the beginning and end of successive soccer seasons, to better program their strategies throughout the training cycle.

Surprisingly, after a short period of training cessation, players experienced a significant enhancement in CMJ performance. Similarly, Santos and Janeira [21] reported significant increases in vertical jump performance after 4 weeks of detraining in adolescent male basketball players. On the other hand, Caldwell and Peters [22], observed significant reductions in jumping height when comparing results collected at the end of the competitive season with data collected after 3 months, at the beginning of the next season (i.e., before pre-season). Based on these results, it is possible to infer that, although long-term detraining (i.e., >4 weeks) may negatively impact jump performance, shorter periods of detraining (i.e., ≤4 weeks) can positively affect this neuromechanical ability in team-sport players. A possible explanation for this phenomenon might be related to the impact of the fitness fatigue paradigm [23]. Here, following short-term training cessation, the fatigue accumulated over the season may dissipate, allowing the potentiation effects of previous training to be revealed, which results in a net increase in some specific physical qualities [23,24]. Furthermore, it has been suggested that higher volumes of soccer training can impair certain neuromuscular capabilities [25]; as a result, when training loads decrease, improvements in jumping ability could be expected. Soccer coaches should consider elaborating their training routines under this perspective, as general preparation phases may not be required (at least in relation to explosive capabilities) after short periods of total training cessation [2,4]. This may also have implications for in-season training design, where a far greater degree of fatigue is usually present, thus requiring a greater manipulation of training variation to attain peak performance.

Contrary to jump performance, no significant changes were observed in strength and speed capabilities (assessed by leg-press 1RM and 10 m sprint velocity). It is well established in the literature that maximum strength and sprint velocity are closely interrelated, with this correlation being stronger at shorter distances (i.e., <20 m), during the early acceleration phases of sprinting [26]. At this stage, athletes need to apply substantial amounts of force at lower velocities onto the ground, in order to overcome the inertia and accelerate their bodies forward as quickly as possible [27,28,29]. Conversely, at top-speed phases, higher contraction velocities are required and, hence, the contributions of the elastic components and stretch-shortening cycle to sprint velocity are increased [27,28]. This mechanical relationship may be confirmed when examining the strong associations regularly found between CMJ and sprint velocity at longer distances (i.e., >20 m) [28,30], in athletes from different sport disciplines. Since a significant increase was only observed in jump performance, it is also expected that the lack of changes in maximum strength would lead to similar (and non-significant) variations in short sprint velocity. However, it is crucial to emphasize that players maintained their initial strength and speed levels (i.e., tests conducted at the end of the soccer tournament) throughout the 26 days of detraining. These findings reinforce the notion that soccer players do not necessarily need to develop the so-called “strength foundation phase” before implementing sprinting-specific workouts, at least after shorter periods of total training cessation (i.e., ≤4 weeks) [2,4,6,31].

This study is limited by its descriptive design. In addition, the study took place between two successive competitions after short-term detraining. Thus, it is not clear whether longer periods of detraining (i.e., >4 weeks) would evoke equivalent responses in these athletes. Moreover, it is not possible to confirm if more experienced soccer players (i.e., senior category) would exhibit similar tendencies after being exposed to a short period of total training cessation. Another issue is that we did not evaluate changes in physical performance over the entire competitive season, which would be important for comparing the effects of detraining with other training phases. Nevertheless, these outcomes are of practical importance for soccer coaches and sport scientists, who always have limited time, within congested fixture schedules, to prepare their athletes. According to these results, as soccer players are capable of maintaining their strength, speed, and power capabilities throughout these short-term periods, it might not be necessary to program a “general preparation phase” in the early stages of periodization (at least after detraining phases ≤ 4 weeks). Future studies should examine the effects of short-term detraining in professional soccer players, as well as investigate the potential effects of total training cessation on other physical performance measures (e.g., change of direction speed, and aerobic-based capacities).

## 5. Conclusions

We observed that a short period of training cessation (i.e., 26 days) between two successive official tournaments was capable of promoting a significant increase in the CMJ height without inducing negative effects on the strength and speed capabilities of under-20 soccer players. Soccer coaches and sport scientists should be aware of these opposite changes to program more effective training strategies after short-term detraining. These chronic responses have crucial implications for retraining and training prescriptions, especially at the beginning of subsequent training cycles (e.g., soccer pre-seasons), when soccer players usually have a limited time to properly develop their physical, technical, and tactical skills.
